# Development of a Machine Vision Method for the Monitoring of Laying Hens and Detection of Multiple Nest Occupations

**DOI:** 10.3390/s18010132

**Published:** 2018-01-05

**Authors:** Mauro Zaninelli, Veronica Redaelli, Fabio Luzi, Malcolm Mitchell, Valentino Bontempo, Donata Cattaneo, Vittorio Dell’Orto, Giovanni Savoini

**Affiliations:** 1 Department of Human Sciences and Quality of Life Promotion, Università Telematica San Raffaele Roma, Via di Val Cannuta 247, 00166 Rome, Italy; 2 Department of Veterinary Medicine, Università degli Studi di Milano, Via Celoria 10, 20133 Milan, Italy; veronica.redaelli@unimi.it (V.R.); fabio.luzi@unimi.it (F.L.); 3 Animal & Veterinary Sciences, Scotland’s Rural College, Roslin Institute Building, Easter Bush, Midlothian, Edinburgh EH9 3JG, Scotland, UK; malcolm.mitchell@sruc.ac.uk; 4 Department of Health, Animal Science and Food Safety (VESPA), Università degli Studi di Milano, Via Celoria 10, 20133 Milan, Italy; valentino.bontempo@unimi.it (V.B.); donata.cattaneo@unimi.it (D.C.); vittorio.dellorto@unimi.it (V.D.); giovanni.savoini@unimi.it (G.S.)

**Keywords:** double nest occupation, infrared camera, imaging analysis, image pattern matching, laying hens’ performance and behavior

## Abstract

Free range systems can improve the welfare of laying hens. However, the access to environmental resources can be partially limited by social interactions, feeding of hens, and productivity, can be not stable and damaging behaviors, or negative events, can be observed more frequently than in conventional housing systems. In order to reach a real improvement of the hens’ welfare the study of their laying performances and behaviors is necessary. With this purpose, many systems have been developed. However, most of them do not detect a multiple occupation of the nest negatively affecting the accuracy of data collected. To overcome this issue, a new “nest-usage-sensor” was developed and tested. It was based on the evaluation of thermografic images, as acquired by a thermo-camera, and the performing of patter recognitions on images acquired from the nest interior. The sensor was setup with a “Multiple Nest Occupation Threshold” of 796 colored pixels and a template of triangular shape and sizes of 43 × 33 pixels (high per base). It was tested through an experimental nesting system where 10 hens were reared for a month. Results showed that the evaluation of thermografic images could increase the detection performance of a multiple occupation of the nest and to apply an image pattern recognition technique could allow for counting the number of hens in the nest in case of a multiple occupation. As a consequence, the accuracy of data collected in studies on laying performances and behaviors of hens, reared in a free-range housing system, could result to be improved.

## 1. Introduction

Free range systems can improve the welfare of laying hens [1]. In these no-cage housing systems, hens can move freely and manifest their natural behaviors. Nevertheless, some problems that can reduce their welfare and productivity can happen. For example, the access to environmental resources can be partially limited by social interactions [2]. As a consequence, the feeding of hens can be not stable and their productivity can result to be lower [3]. In addition, damaging behaviors, such as cannibalism and piling, or negative events, such as bone fractures, parasites, and diseases can be observed more frequently than in conventional housing systems [4].

In order to reach a real improvement of the hens’ welfare, by the use of these alternative rearing systems, the study of their laying performances and behaviors is necessary. Through these data, as collected in a real scenario, it can be possible to: (1) select a commercial strain that shows to be more suitable for this kind of rearing systems [5,6,7]; (2) improve the design of these alternative systems in order to make them appropriate to the needs of hens [2]; and (3) identify a set of best practices in order to assist farmers in the management of these housing systems [7].

With this purpose, many systems have been developed for the monitoring of laying hens in a free range environmental [8,9,10,11,12,13,14,15,16,17,18,19,20,21,22,23]. Within all of these systems, the Funnel Nest Box (FNB), as developed by Thurner et al. [8], is the most cited device. This system is based on the use of Radio Frequency Identification (RFID) technology. An RFID antenna is positioned in the floor of the nest. A visit of the nest is recorded through the reading of a transponder tightened to the hen’ leg. The duration of a nest visit is continuously updated until the transponder is in the reading area of the RFID antenna. Each egg laid is collected in a tube and detected by a sensor. Through the information of the hen’ transponder and egg laid, an “egg-to-hen” assignment can be built and recorded by the system. All of these data, combined with the number of nest visits and the duration of the nest occupations, allow for the system to figure out the behavior and productivity of the hens of the flock. Another system for the monitoring of laying hens was developed by Burel et al. [11]. Also, this system is based on the use of the RFID technology, but, in this case, an antenna is positioned at the nest entrance and each hen wears two transponders, one in its neck and the other one in its leg. Through the sequence of transponder readings, the system detects the entry or the exit of a hen from the nest. The difference between the two timestamps allows to calculate the nest visit durations. Furthermore, each egg laid is collected through an “egg-collector”, detected by an “egg-detector” and linked to the corresponding hen through the merging of all these data.

When a system for the monitoring of laying performances and behaviors of hens is adopted, a double occupation of the nest can occur up to the 32% of the nest occupations, depending on the number of hens per single nest that are used for the experiment [18]. These events can affect the correctness of data collected and therefore the accuracy of the monitoring system. Different strategies have been developed to limit these unfavorable cases. For example, in the system developed by Burel et al. [11], the nest is narrowed in order to prevent that more than one hen is in the nest in the same time. In the system developed by Marx et al. [10], a trap device is adopted in order to block the nest entrance when a hen is in the nest, avoiding that other hens may enter. A similar device is used in the FNB, as developed by Thurner et al. [8]. The floor of the nest can move. When a hen enters in the nest, the floor rotates. The rotation locks the trap and it avoids that other hens can enter in the nest while it is already occupied. However, all of these strategies do not completely avoid a double occupation of the nest. A concurrent entry, of the nest, of more than one hen, can always happen. Furthermore, the presence of trap devices can modify the behaviors of hens [11], and thus limits the accuracy of data collected. Therefore, a different strategy should be selected in order to manage these events when they occur.

In this direction, an improved FNB has been developed and described by Icken et al. in 2013 [18]. This system has a double tilting floor. When a hen enters in the nest, the floor makes a first rotation in order to lock the nest entrance. If two hens enter the nest in the same time, the resulting weight on the nest floor is greater. As a consequence, the nest floor is forced to make a second rotation that activates a sensor and a corresponding alarm. In this way, the system is able to detect a double occupation of the nest and thus improves the accuracy of data collected. However, in field tests, this system showed to detect the 60% of the double nest occupations occurred [24]. Furthermore, it requires many mechanical and electronic components for each nest box that can increase costs, and thus to limit experiments curried out with a large flock.

A different solution to detect a double nest occupation through a system for the monitoring of laying performances and behaviors of hens has been tested by our research group in 2015 [25]. This “nest usage sensor” is based on the use of a common webcam and collects images, from the black colored interior of the nest, when one or more hens enter in the box. The algorithm, as implemented by a dedicated software routine, requires to count the number of colored pixels of each image. When this number is higher than a specific threshold, the sensor sends an alarm to the monitoring system. Results obtained in field tests showed that the sensor was able to detect up to 94.6% of the double nest occupations occurred during an oviposition [25]. Nevertheless, some false-negative cases were observed mainly due to low light conditions and/or shadows that affected the count of colored pixels in the images evaluated. In addition, when more than two hens entered in the nest, the sensor detected a double nest occupation, but no information about the real number of hens in the nest were available. In order to overcome these issues some different technologies should be considered, such as the infrared (IR) spectrum and the image pattern recognition.

A body, which has a temperature higher than the absolute zero, emits electromagnetic radiation in the IR spectrum. The relationship between the energy emitted, the wave length of this radiation and the temperature of the body is mathematically described by the Planck, Wien, and Stefan-Boltzmann laws [26]. This radiation can be detected through a thermo-camera and used to build a thermografic image where the intensity of each pixel, or of a set of them, is proportional to the originally temperature of the body observed. Furthermore, when the body has an higher temperature than the background where it is positioned, its shape, in the thermografic image, can result to be easily identifiable despite low light conditions, dark, or when possible shadows are visible. Therefore, the adoption of a thermo-camera could be a valid support to overcome the above cited issues in the detection of a double nest occupation. Furthermore, a thermografic image could be investigated performing image pattern recognition in order to detect, in automatic, a specific object inside it. This technique of analysis is generally used to carry out quality controls of industrial processes, even though different examples can be observed in many applications of machine vision. Image pattern recognition can be performed when a template is identified. This is a physical model of the object that is necessary to search in the image evaluated. More than one object can be in the same image and all objects can be detected by the procedure even though they are rotated, in part distorted, or they have different dimensions due to a different distance from the shooting point. This technique, therefore, could be a possible way to figure out how many hens are in the nest in case of a multiple occupation.

Infrared spectrum and image pattern recognition are technologies that have been partially investigated in poultry researches. Thermografic images, in detail, have been mainly tested to improve poultry’ welfare [27,28,29]. Yahav et al. [30], for example, evaluating data from a thermo-camera, measured the body surface temperature of chickens in order to calculate heat loss by radiation and convection. The authors proved that climate-control systems, welfare, and performance of the flocks could be improved by the evaluation of physiological state of chicken, in different environmental conditions, performed through thermografic images. Ferreiera et al. [29], studying the loss of sensible heat in young broilers fed with different dietary energy levels found that IR technology allowed for improving productivity and welfare of animals through the recording of the variations of the young broilers’ surface temperature. Baracho et al. [27], measuring the broilers’ surface temperature, through the use of thermografic images, in relationship with the heat distribution of their houses found important variations due to the week of grow-out and sector of broilers’ house. In this way, the authors were able to demonstrate that broilers were suffered a thermal distress. Also, techniques of image pattern recognition have been studied in poultry researches by some authors with interesting results [31,32,33,34]. For example, Nakarmi et al. [32] used some image processing procedures to identify and track hens in the experimental pen where a system, based on a three-dimensional (3D) vision camera and a grid of twenty RFID antennas, were installed. Through these image procedures, the authors were able to study the behavior of hens and to analyze the effects due by different housing designs and management. Wang et al. [33], instead, evaluating different software algorithms to track a singular hen in a flock of laying hens, found that an algorithm, based on the Hybrid Support Vector Machine, could be a valid method to study the behavior of hens without the need of additional sensors. Kashiha et al. [34], investigating the performance of image analysis found that a system, made by four cameras mounted on the top of four interconnected sections of an experimental cage, was able to track laying hens with good results if compared with the human ability to detect animals. Finally, also, our research group tested these technologies in a commercial organic egg production farm [35,36]. The resulting monitoring system allowed for detecting the possible presence of hens in the inner part of an housing system permitting to project possible ozone treatments, in a safe mode both for animals and workers, and consequently, to achieve a possible reduction of levels of atmospheric ammonia and bacterial load in eggs production farms, with a particular focus on those that produce organic eggs. However, even though these technologies have proved to be useful in the monitoring of laying hens activities, they have never been considered for the developing of a “nest usage sensor” able to count the number of hens in a nest, and thus to improve the accuracy of data collected in studies on laying performance and behavior of hens.

The aim of the present study was therefore the development and testing of a “nest usage sensor” based on the use of a thermo-camera and the performing of image pattern recognition. The targets to reach with this new device were: a better detection of double nest occupations and the count of the number of hens that were in the nest in case of a multiple occupation. These targets were selected in order to improve the accuracy of the data collected in the monitoring of laying performances and behaviors of hens reared in a free-range housing system.

## 2. Material and Methods

### 2.1. The Experimental Housing System

**Figure 1:**
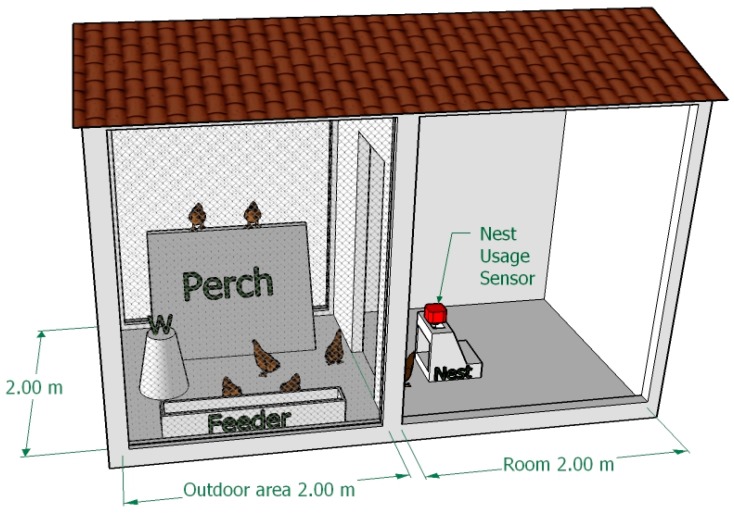
Dimensions and of the experimental housing system set-up. The positioning of the experimental nesting box and of some main components, is reported. The water dispenser is represented by the symbol “W”.

The developed “nest-usage-sensor” was set-up and tested through an experimental nesting system installed in an experimental housing system. This housing system was a free-range system composed by a covered outdoor area of 4.0 m^2^ (2.0 × 2.0 m) and a room with the same dimensions (Figure 1). A water dispenser, a feeder, and a perch was provided to hens in the outdoor area, while the experimental nesting system was positioned in the room, close to the room entrance, as shown in Figure 1.

### 2.2. The Experimental Group of Animals

In field tests, ten laying hens were randomly selected among those reared in a commercial egg production farm located in the North of Italy (Lombardy region). All of the hens were of Institut de Sélection Animale (ISA) brown breed, selected at the age of circa 18 weeks, already reared in group and housed in the experimental housing system one month before the start of the tests.

The number of hens of the experimental group was defined while considering the value cited in the Article 4.1.c (“all systems must be equipped in such a way that all laying hens have at least one nest for every seven hens”) of the EU Council Directive (1999/74/EC-that fix the “minimum standards for the protection of laying hens”) increased of 50% with the scope to have, during the tests, an higher number of double nest occupations since, as it is known, this value is affected by the number of hens per single nest [18].

### 2.3. The Experimental Nesting System

The developed “nest-usage-sensor” was set-up and tested through an experimental nesting system. This system was mainly composed by a single nest box, of sizes 30 cm × 40 cm × 50 cm. It did not have any trap device, positioned at its entrance. Its floor (without any bedding material) had a slope of circa 15 degrees in order to facilitate the exit of each egg laid through an “egg exit hole” positioned in the bottom of the nest. In the back of the nest, a couple of photo cells was mounted in order to detect each egg during its fall in a tube which stored the whole production of each day, in the order of laying. Photo cells had a diameter of 4 cm, global sizes of 5.5 cm × 5.5 cm × 7.2 cm, a power supply of 12 V, a sampling rate of 850 Hz ± 10%, and were connected to an Analog to Digital (AD) conversion board (National Instruments, Austin, TX, USA–model: PCI MIO 16E 4) in order to collect their signals from a PC.

Some days before the start of the tests, a transponder (ISO 11784/85 compliant) was tightened to each hen’ leg. According to scientific literature [37,38,39], no change in the hens’ behavior was observed due to the wearing of the transponders. During the tests, hens’ transponders were read by an RFID system composed by a stationary ISO reader provided by Fasthink (Fasthink Srl, Mezzago, Italy–model: BlueBox Gen2 LF) and an antenna, of trapezoidal shape, embedded in the floor of the nest.

**Figure 2:**
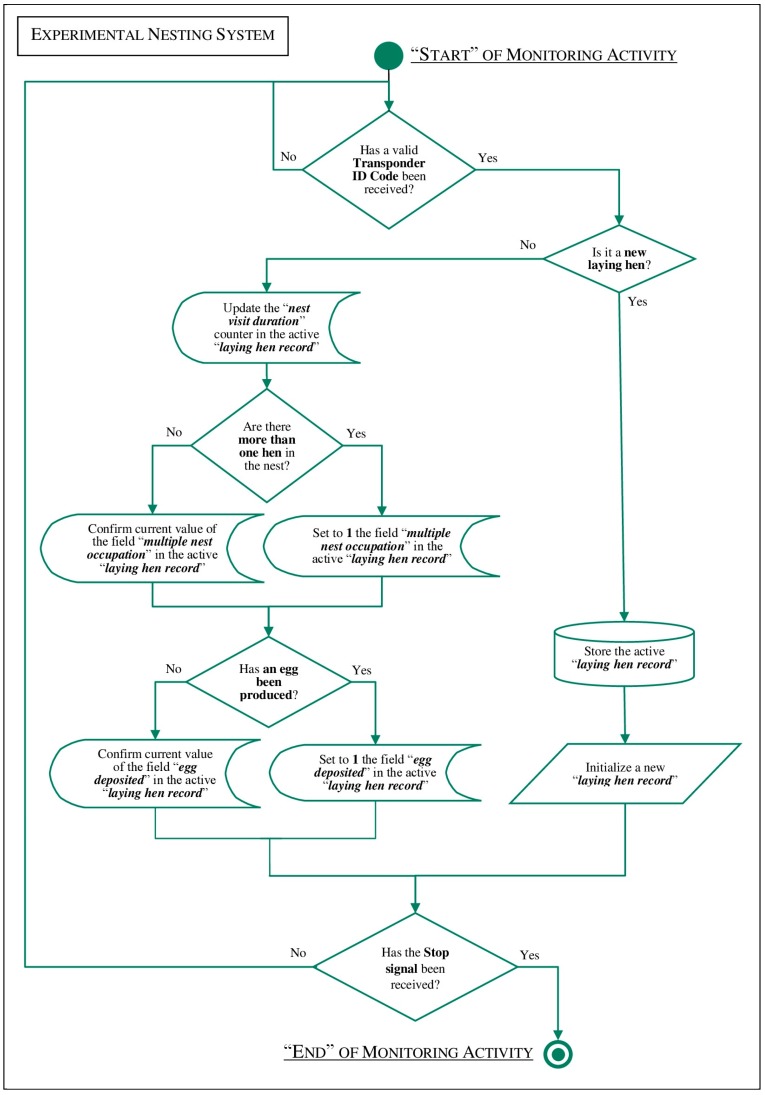
Flow diagram of the software application developed for the experimental nesting system.

Hardware components used in the experimental nesting system were controlled by dedicated software application developed with LabVIEW (National Instruments, Austin, TX, USA–version 8.5). The tasks performed by the software application are described in the flow diagram reported in Figure 2. As it is shown, when a new hen is identified by its transponder a new “laying hen record” is created [40]. If the hen stays in the nest, its transponder is repetitively read by the RFID system and the record field “nest visit duration” is continuously updated. When a multiple nest occupation is detected by the “nest-usage-sensor”, the record field “multiple nest occupation” is set-up. If an egg is laid and detected by the couple of photo cells during its fall in the collection tube, the record fields, called “egg deposited” and “deposition time”, are set-up. Finally, if the hen stays in the nest for more time after to have laid an egg, the record field “nest visit duration” continues to be updated. Only when a new hen is identified through its transponder, the current “laying hen record” is stored (by a log text file) and a new one is initialized [41] with all of its record fields reset to zero. Of course, all values recorded during a monitoring session are shown to users by a dedicated graphical user interface.

The experimental nesting system also acquired images from the nest interior in order to provide a feedback on the correctness of data collected during the tests carried out. These images were single snapshots collected by a commercial Universal Serial Bus (USB) web-cam (HAMA, Monheim, Germany–model: AC-150), of sizes 5 cm × 1.5 cm, mounted on the top of the nest close to the “nest-usage-sensor”. Images were acquired in the “.bpm” format, with a resolution of 320 × 240 pixels, a sampling rate of 1 s and a time stamp added in the left upper corner of each image. Furthermore, images were used to build video recordings in the “.avi” format, compressed by the filter “Microsoft Video 1”, and with a picture sampling rate of 1 Hz. During the tests, all of the images and video recordings were stored in an external memory using dates and time stamps to name each file [42]. The range of time in which snapshots were collected started when at least one hen was in the nest and stopped when no hens were detected inside it. Video recordings were instead acquired every day, starting from the opening of the nest to hens (at 6:00 a.m.) and stopping before the manual closing of the nest (at 18:00 p.m.).

### 2.4. The “Nest-Usage-Sensor” Developed

**Figure 3:**
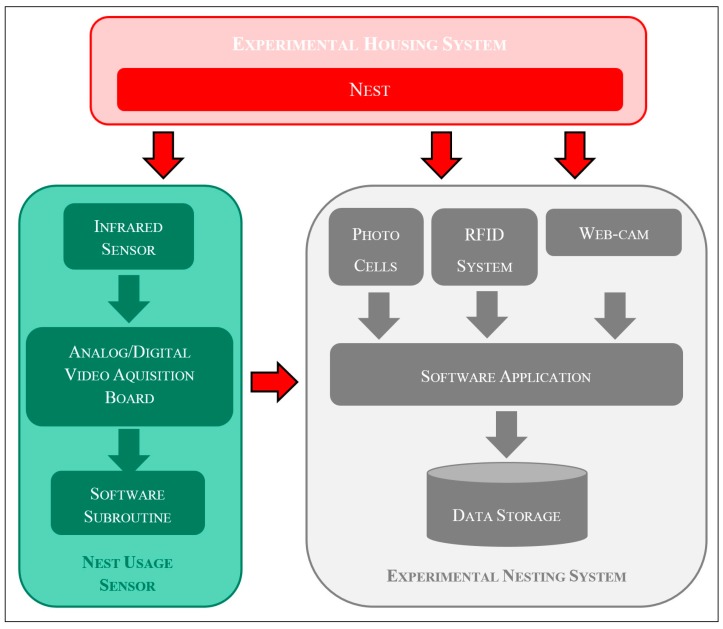
Functional schema of the hardware and software components of the “nest usage sensor” with their connections to the other main components of the experimental nesting system.

The “nest usage sensor” was basically a machine vision system composed by: a thermo-camera for the acquisition of thermografic images; an AD video conversion board; and, a dedicated software subroutine for the automatic elaboration of images acquired. The functional schema of these hardware and software components is provided in Figure 3.

The thermo-camera (Nippon Avionics Co., Tokyo, Japan–model: Thermo GEAR-G120) was an uncooled (microbolometer) focal plane array detector with a resolution of 320 × 240 pixels. Its accuracy was ±2 °C with a sensitivity of 0.04 °C (at 30 °C), while its sizes were 21.2 cm × 7.5 cm × 13.8 cm. Furthermore, it had an analog video output in order to be connected to an external monitor, a common function available in many commercial thermo-cameras.

An AD video conversion board (ROXIO, Santa Clara, CA, USA) was used in the system in order to provide, in a digital format, the grey scale thermografic images received from the video output of the thermo-camera.

**Figure 4:**
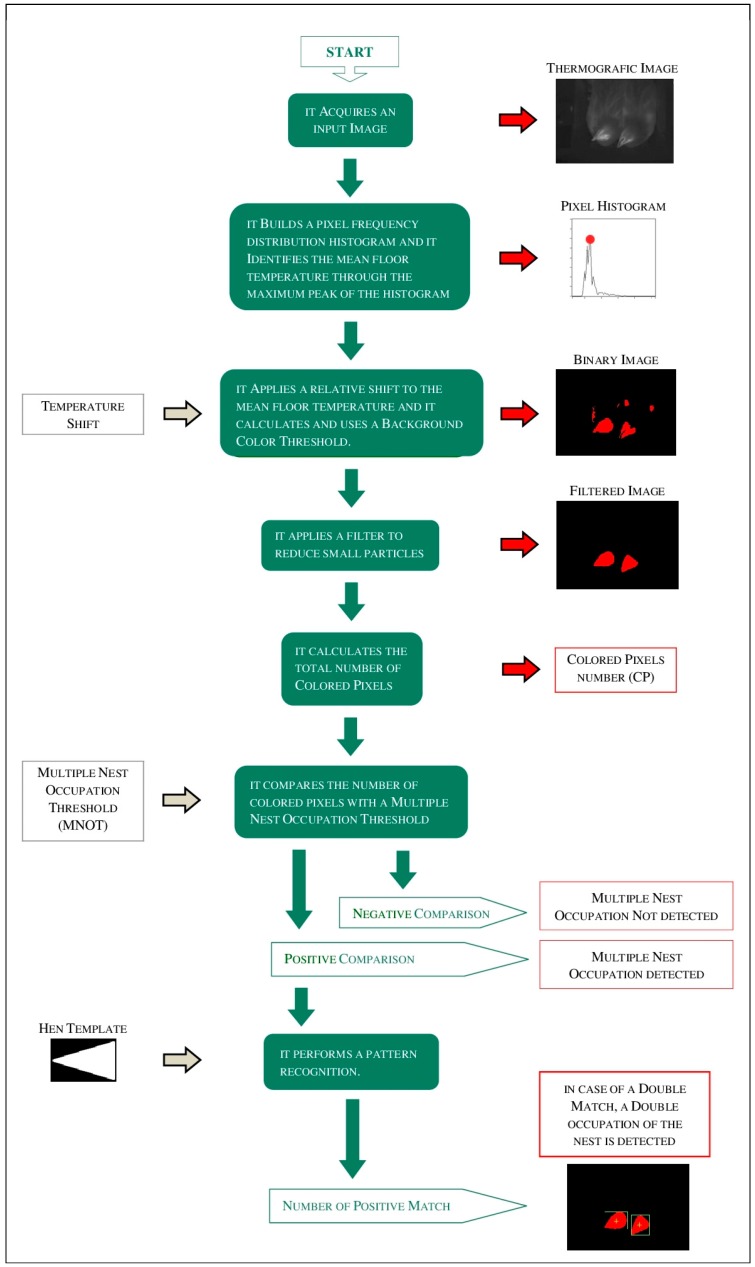
Activity diagram of the software subroutine of the “nest-usage-sensor” developed.

A dedicated software subroutine was developed in order to evaluate the thermografic images acquired. It was developed using: LabVIEW (NI–version: 8.5), the Vision Acquisition Software (NI–version: 2009), and the Vision Development Module (NI–version: 2009). The subroutine, on each grey scale image received from the AD video conversion board, performed some specific activities (Figure 4). In details:

- it calculated an histogram with the frequency distribution of the pixel intensities;
- it calculated the “mean floor temperature” of the room when considering that: (a) the thermo-camera is pointed down; (b) to each pixel intensity of the thermografic image corresponds a specific measured temperature; and (c) the histogram generally has a normal distribution where the mean value corresponds to the background intensity;
- it added a defined shift to the calculated “mean floor temperature” and it defined a “Background Color Threshold” (BCT) converting the value obtained in a grey scale color;
- it created a binary image giving a color to all grey scale colored pixels that overcame the BCT;
- it improved the quality of the binary image reducing the number of small “particles” through the application of an image filter;
- it counted the number of “Colored Pixels” (CP);
- it compared the number of CP with a “Multiple Nest Occupation Threshold” (MNOT), a value that is proportional to the “visible” area of more than one hen in the nest;
- when the number of CP overcame the MNOT, it set-up to 1 the field “Multiple Nest Occupation” (MNO) and it performed a pattern recognition, using a defined “hen template”, in order to determine how many hens were in the nest at the same time; and,
- when the number of CP did not overcome the MNOT, it set-up to 0 the field MNO.

In order to identify the number of hens that were in the nest, a pattern recognition procedure was carried out by the software subroutine of the “nest-usage-sensor”. The procedure calculated the Normalized Cross-correlation between the binary image obtained and an “hen template” previously set-up [43,44]. Normalized Cross-correlation can be considered as a particular case of correlation that can be described by the following Formula (1):

(1) $$C\left(i,\;j\right)=\overset{V-1}{\underset{x=0}{\sum }}\overset{U-1}{\underset{y=0}{\sum }}t\;\left(x,y\right)\;p\left(x+i,y+j\right)$$

where p(x,y) is the image evaluated of sizes W×Z; t(x,y) is a template of sizes U×V with U≤W and V≤Z; i=0, 1, …, W−1; j=0, 1, …, Z−1; and, the summation is obtained taking into consideration the overlapping between the image p and the template t. In practice, the procedure of correlation requires to shift t within p and it evaluates the value of C(i,j) when considering the region where the template t overlaps the image p. Thus, each pixel of t is multiplied for the corresponding pixel of the picture p that it overlaps. Each result is summed over all the pixels of template t. The point where the function C(i,j) is maximum, is the place of the image evaluated where the template t can be found.

However, pixel intensity changes of the images evaluated can affect the counts of the correlation procedure, and thus its sensitivity. To limit this negative effect, a normalized cross-correlation can be considered, as it was done in this study, according to the following Formula (2):

(2) $$R\left(i,j\right)=\frac{\sum_{x=0}^{V-1}\sum_{y=0}^{U-1}\;\left[t\left(x,y\right)-\bar{t}\right]\;\left[p\left(x+i,\;y+j\right)-\bar{p}\left(i,j\right)\right]}{{\left\{\sum_{x=0}^{V-1}\sum_{y=0}^{U-1}\;{\left[t\left(x,y\right)-\bar{t}\right]}^{2}\right\}}^{\frac{1}{2}}\;{\left\{\sum_{x=0}^{V-1}\sum_{y=0}^{U-1}\;{\left[p\left(x+i,y+j\right)-\bar{p}\left(i,j\right)\right]}^{2}\right\}}^{\frac{1}{2}}}$$

where: $\bar{t}$ is equal to the mean value of the pixel intensities of the template t and can be evaluated only one time; and, $\bar{p}$ is the mean value of the pixel intensities of a region determined by the position of the template *t*. The resulting value (R) is always in a range from −1 to 1 and is not affected by any changes of the pixel intensities of both images (p and t). Furthermore, when the object to detect has a different orientation respect the template t, a new procedure can be performed rotating the image template.

### 2.5. The Design of the Performed Tests

All of the tests were conducted in a month (i.e., four weeks–2 October to 29 October). Data collected in the first week were used to perform a set-up procedure of the “nest-usage-sensor”, while data stored in the other weeks were considered to evaluate its performances.

The setup procedure of the “nest-usage-sensor” was carried out in two steps (A and B) using a dedicated version of the experimental nesting system software application able to work off-line on “.bmp” files. With this software application, a subset of thermografic images (*n* = 20,000), acquired by the thermo-camera, was analyzed. This subset of images included 10,000 single nest occupations (randomly selected among 43,698 nest occupations manually classified by a researcher as a single occupation) and 10,000 multiple nest occupations (randomly selected among 20,849 multiple occupations of the nest). In order to build the BCTs, three shifts of the mean floor temperature, equal to 1, 3, and 5 Celsius degrees, were considered in both steps of the setup procedure. These shifts were chosen on the basis of our previous experiences [35,36] and considering what scientific literature suggests [27,29,30,45], as possible body temperatures that should result in a thermografic image of a hen.

#### 2.5.1. Setup Procedure of the “Nest-Usage-Sensor”: Step A

For each shift of the mean floor temperature, and thermografic image, the resulting number of CP was calculated. The significance of the mean values of CPs between real cases of single nest occupation and real cases of multiple occupation were checked performing an ANOVA procedure (statistical analysis tool: R ver. 3.3.0 [46]-package *stats* ver. 3.3.0, procedure *aov* [47]).

The values of CP were studied also to discriminate a possible case of single occupation from a possible case of multiple occupation using different cutoff levels; and, each discrimination was compared with the real number of hens that were in the thermografic image. The result of each comparison was classified as: true positive (TP), when multiple occupations of the nest were correctly detected; false positive (FP), when a multiple occupation was detected but only one hen was in the nest; true negative (TN), when a single occupation of the nest was correctly detected; and, false negative (FN), when a single occupation on the nest was detected, while more than one hen was in the nest. When all of the comparison results were classified, the performances showed by the “nest-usage-sensor” for each shift of the mean floor temperature, and used cutoff level, were calculated as: sensitivity and specificity, in accordance with the following Equations (3) and (4):

(3) $$\mathrm{Sensitivity}\left[\%\right]=\frac{\mathrm{TP}}{\mathrm{FN}+\mathrm{TP}}$$

(4) $$\mathrm{Specificity}\left[\%\right]=\frac{\mathrm{TN}}{\mathrm{FP}+\mathrm{TN}}$$

For each shift of the mean floor temperature, the cutoff level that allowed to reach a sensitivity of at least 80% was selected and used as MNOT value in the elaborations that followed. A reference of sensitivity of 80% was chosen because it was supposed to be a reasonable level of the human ability to detect a hen in a thermografic image.

In this step of the setup procedure also the templates, as necessary for the following pattern recognitions, were studied. They were geometric shapes selected, for each shift of the mean floor temperature, on the basis of the thermografic imprints of a hen, and, according to our previous tests [35]. In detail, when the BCTs were built with a temperature shift of 1 °C, all of the particles in each thermografic image were identified and for each of them an equivalent ellipse, with its minor and major axes, was calculated. When the BCTs were built with a temperature shift of 3 or 5 °C, a similar procedure was performed but in these cases a bounding rectangle, with its long and short side, was calculated. At the end of these elaborations, three templates were selected. For the temperature shift of 1 °C, the template had an elliptical shape (because proportional to the whole body of a hen) and sizes equal to the mean values of the minor and major axes of the equivalent ellipses previously calculated. For the temperature shift of 3 or 5 °C, each template had, instead, a triangular shape (because proportional to the head of a hen) and sizes (in terms of heights and bases) equal to the mean values of the long and short sides of the bounding rectangles previously calculated. These templates were used in the step of the setup procedure that followed.

#### 2.5.2. Setup Procedure of the “Nest-Usage-Sensor”: Step B

During this step of the setup procedure, the same subset of thermografic images was evaluated. The same shifts of the mean floor temperature were investigated and for each of them, a specific MNOT and template, within those identified, were used to configure the software subroutine of the sensor. Procedures of image pattern recognition were carried out and used to discriminate a possible case of single occupation from a possible case of multiple occupation. Each discrimination result was compared with the real number of hens that were in the thermografic image and finally classified as: TP, FP, TN, and FN. At the end of all of these classifications, the performances achieved by the sensor for each shift, of the mean floor temperature, were calculated in terms of: sensitivity and specificity, in accordance with the equations reported in the previous paragraph (3)–(4).

At the end of these elaborations, the setup procedure of the “nest-usage-sensor” was concluded and the mean floor temperature shift, with the corresponding MNOT and template features, which showed the best detection performance, was identified and used for the tests that followed.

#### 2.5.3. Performance Evaluation of “Nest-Usage-Sensor”

In the evaluation phase of the “nest-usage-sensor”, the laying data and behavior of hens were registered by the experimental nesting system. Hens that entered the nest were identified by the RFID system through their transponders. Eggs laid, rolling out from the nest, were detected by the egg-sensor and for each of them; the corresponding “egg-to-hen” assignment was built and stored by the experimental nesting system. Multiple occupations of the nest were also monitored by the “nest-usage-sensor” and recorded in a log file. Every day of test, after the manual closing of the nest, a researcher picked up the eggs from the collection tube. According to the data provided by the log file, he/she marked each egg with the ID of the corresponding hen. Possible differences between data provided by the log file and the sequences of eggs picked up from the collection tube, were archived. Furthermore, the video recordings of the nest interior, which were acquired daily, were analyzed in order to control data provided by the “nest-usage-sensor”. Its performances were therefore calculated in terms of sensitivity and specificity.

During field tests, the lenses of the thermo-camera and of the web-cam were checked and when necessary, they were cleaned by a researcher to guarantee that high quality images were acquired. A similar procedure was performed also for photo cells in order to limit possible faults of the egg-sensor.

## 3. Results

When field tests were performed, room temperature was always in the range 13–21 °C, with a mean value of circa 18 °C. Some shifts of the mean floor temperature, adopted to build the BCTs, were investigated. They were shifts of 1, 3, and 5 °C. A set of thermografic images was used to setup the “nest-usage-sensor”. For each of them, the resulting number of CP was calculated according to each shift of the mean floor temperature evaluated. The significance of the mean values of CPs between real cases of single and multiple nest occupation were checked performing an ANOVA statistical procedure. Results obtained are reported in Table 1.

Mean values of CPs, calculated on thermografic images of single and multiple occupations, showed a significant difference for each shift of the mean floor temperature investigated, as reported in Table 1. Therefore, possible detection performances, at different cutoff levels, were studied in terms of sensitivity and specificity reached by the sensor. Results obtained, imposing sensitivity to of at least 80%, are reported in Table 2, for each temperature shift considered.

In the same step of the sensor setup procedure (step A), also the templates, necessary to perform the image pattern recognitions, were investigated for each shift of the mean floor temperature studied. They were templates of elliptical or triangular shape. The results found are shown in Table 3. Furthermore, confidence intervals and significance of the means, evaluated by a *t*-Test of Student (statistical analysis tool: R ver. 3.3.0 [46]-package *stats* ver. 3.3.0, procedure *t*-test) are also reported in the table.

In a second step of the sensor setup procedure (step B) carried out on the same set of thermografic images, the detection performances of the “nest-usage-sensor” were calculated always in terms of sensitivity and specificity. The sensor performances were evaluated for each mean floor temperature shift investigated, adopting: (a) as MNOT value, a cutoff level that was in accordance with the results reported in Table 2; and (b) for the image pattern recognition procedure, a template that was in accordance with the results reported in Table 3. The results that were observed are reported in Table 4.

The best detection performances, in terms of sensitivity, and, specificity, was reached by the “nest-usage-sensor” with a shift of the mean floor temperature equal to 5 degrees, as shown in the table. As a consequences, in the field tests that followed, the “nest-usage-sensor”, and the experimental nesting system, were setup with the parameters: (a) a shift of the mean floor temperature of 5 °C for the calculation of the BCTs; (b) a MNOT equal to 796 pixels in order to classify possible cases of multiple occupation of the nest; and (c) a template of a triangular shape and sizes of 43 × 33 pixels for the performing of the pattern recognition procedures.

In field tests, which were carried out to evaluate the detection performances of the “nest-usage-sensor” in a real scenario, data collected by the experimental nesting system were considered. During field tests, eggs productivity of the hens of the experimental group was not different in mean if compared with the average farm productivity. In details, the eggs laid by hens were 184. Of these eggs, 178 (96.7% of the total amount of eggs laid) were deposed in the nest were while six (3.3%) were deposed outside of the nest. The “egg-to-hen” assignments done by the experimental nesting system were 122 (68.5% of the total amount of eggs laid inside a nest), while for 56 eggs (31.5%), the assignment of the egg to a specific hen of the experimental group was not possible. The reasons of these failures were: (a) eggs blocked in the nest (two eggs, 1.1%) or in the collection tube (by broken eggs-two eggs, 1.1%); (b) eggs not detected by the photo cells (one egg, 0.6%); and (c) eggs that were uncertain (51 eggs, 27.2%), because a multiple occupation of the nest was been detected by the sensor.

**Figure 5:**
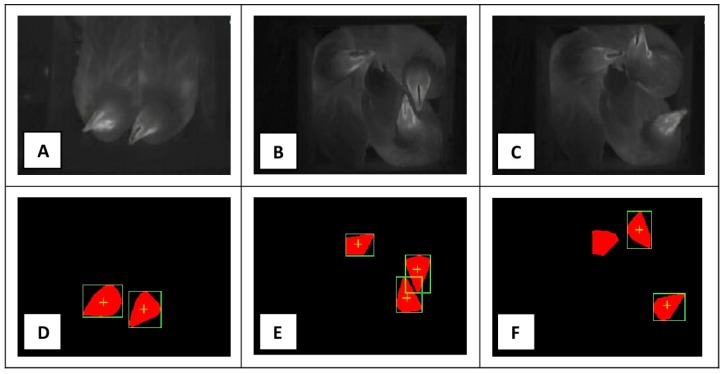
The pictures (**A**–**C**) are thermografic images acquired by the “nest-usage-sensor” from the nest interior; The pictures (**D**–**F**) are the results of the image pattern recognition procedures performed by the sensor having as input the images (**A**–**C**); Picture (**D**) is an example of a true-positive case considering the detection of a multiple or double nest occupation and, in the same time, an example of a true-negative case if the detection of a triple nest occupation is evaluated; Picture (**E**) is an example of a true-positive case, when considering the detection of a multiple or triple nest occupation, and an example of a true-negative case for the detection of a double nest occupation; Finally, picture (**F**) is an example of a true-positive case about the detection of a multiple nest occupation; of a false-positive case for the detection of a double nest occupation and of a false-negative case if the detection of a triple nest occupation is taken into consideration.

In a final step of the sensor evaluation, all ovipositions were studied. Data stored in the log files were compared with video recordings acquired daily from the nest. The results of all these comparisons were classify as: TP, FP, TN, and FN (Figure 5). Results obtained are shown in the following table (Table 5).

On the basis of the results reported in Table 5, sensitivity and specificity of the “nest-usage-sensor” were calculated and considered as its performances in the detection of a multiple occupation of the nest. In details, they were: a sensitivity of 95.7% and a specificity of 95.4%. Therefore, only two cases of multiple occupation of the nest, happened during field tests, were not correctly detected by the sensor. In addition, also its ability to count the number of hens that were in the nest during a multiple occupation was investigated through a similar procedure. The results that were obtained are reported in Table 6.

With the results obtained, and reported in Table 6, sensitivity and specificity of the sensor were calculated. In detail, they were: a sensitivity of 73.8% and a specificity of 94.9% in case of a double occupation of the nest; and, a sensitivity of 80% and a specificity of 94.8% in case of a triple occupation of the nest.

## 4. Discussion

The sensor under test showed an improvement in the detection performance of a multiple nest occupation if compared with a similar sensor that we developed in a previous study [25] (that had a sensitivity of 94.6%, and a specificity of 94.8% vs. a sensitivity of 95.7%, and a specificity of 95.4% reached by the new developed sensor). The previous sensor was based on the evaluation of images acquired in the visible spectrum. A double occupation of the nest was detected when the number of pixels, that had a color different from the nest’ interior, overcame a specific threshold. Possible shadows, due to the sun and/or specific light conditions, showed to affect the reliability of the sensor developed. The present sensor overcame this issue as it was, in general, expected. Thermografic images, in fact, allow for easily recognizing the contour of a body that has a temperature higher than the background where it is positioned. In addition, potential shadows do not generally affect this recognition. This feature was confirmed in our tests, and it was the main reason at the basis of the improvements that were reached by the new “nest-usage-sensor” that was tested.

Furthermore, the previous sensor was not able to identify how many hens were in the nest during a multiple occupation. The present sensor, instead, was specifically developed to achieve this result. This allowed improving the accuracy of data collected. Therefore, with this new sensor, a better knowledge of hen’ behaviors in the use of free range system resources could be reached. Pattern recognition technique, applied to thermografic images, was the main reason of the result obtained. However, also the setup of the monitoring system improved the correctness of data collected. In fact, it required to store a “laying hen record” only when a new hen was detected by the monitoring system, imposing therefore to evaluate more than one thermografic image before classify a case. As a consequence, the global detection performance of the system resulted to be high.

Finally, also this new sensor allowed for detecting a multiple occupation of a nest reaching a good accuracy in the monitoring of laying hens without the need of any further mechanical part, like as example a trap device. The construction of the nest, in recording systems, like the FNB [8], could be—as a consequence—simplified and during field tests, data could be collected without affect the natural behavior of hens. Therefore, a better knowledge of hens behavior, reared in free range hosing systems, could be achieved and better results, in terms of: selection of commercial strain; improvement of the design of these alternative systems; identification of a set of best practices in order to assist farmers in the management of these housing systems, should be obtained, with a possible increase also of the hens’ welfare.

However, some multiple occupations of the nest were difficult to detect. They were due to the number of hens that were in the nest in the same time or by the position of one of them on the border of the nest (i.e., with its head immediately outside of the nest). In these cases, not all the hens were “visible” to the sensor, and thus immediately identifiable. However, a high number of hens in the nest were observed basically in the first days of the test curried out. Therefore, if the experimental nesting system had been available to hens more than a week before the start of the tests, probably these unfavorable events would have been fewer. Furthermore, during the definition of the experimental group, a high density of hens was chosen in order to have a bigger chance to have a multiple occupations of the nest. Therefore, with a lower density of hens in the flock, as required by the EU Council Directive, or a bigger number of nests, these negative events should result to be less frequent. Finally, the position of the sensor could be changed in order to also monitor the entrance of the nest. With this set-up, also the hens that could stay on the border of the nest could be better controlled and detected.

More in general, image analysis, and image pattern recognition, confirmed to be a valid way for the monitoring of hens [35,36]. Furthermore, thermografic images allowed for collecting useful information on the laying performance and behavior of animals. However, to collect thermografic images, a thermo-camera must be used. Commercial thermo-cameras are still costly devices and their performances can be affected by the environmental conditions where they are used. Dust and dirt, which are generally present in the buildings where the hens are reared, can be a possible source of issues. However, some additional technical devices could be adopted in order to limit these possible problems. For example, compressed air could be sprayed on thermo-camera lenses at the beginning of monitoring activities, or at specific interval of times, through a nozzle that was mounted close to the infrared device. This should limit the deposition of dust and/or of dirt. In addition, a commercial device to shield the camera could be adopted. These devices have a cover positioned in front of the thermo-camera lenses. The cover can be moved through a pneumatic system, allowing for acquiring thermografic images, when necessary, and protect lenses in the remaining interval of times. Furthermore, to reduce costs, specific setups of the monitoring system could be considered. For example, a thermo-camera could be mounted on a track and moved around one or two axes. When considering that software subroutine of the “nest usage sensor” can perform one evaluation cycle for each second, it could be used to control more than one nest. Therefore, this solution could allow splitting the cost of a thermo-camera on more nests and consequently to limit the general cost required to build a monitoring system addressed to studies, or to monitor, a large flocks.

## 5. Conclusions

The results obtained in this study proved that the performance of a sensor for the monitoring of laying hens, reared in a free-range housing system, could be improved through the adoption of a thermo-camera and the performing of image pattern recognitions. The evaluation of thermografic images would increase the detection performance of a multiple occupation of the nest and the performing of pattern recognitions, on acquired images, would allow counting of the number of hens in the nest in case of a multiple occupation. As a consequence, the accuracy of data collected in studies on laying performances and behaviors of hens, reared in a free-range housing system, could result to be improved.

## Figures and Tables

**Table 1:** Significance of mean values of Colored Pixels (CPs) between real cases of single and multiple nest occupation for each mean floor temperature shift investigated (i.e., 1, 3, and 5 °C). Significance of mean values has been evaluated performing and ANOVA statistical procedure on data collected.

Shifts of the Mean Floor Temperature (∆*t*-°C)	Mean Values of Colored Pixels in Single Nest Occupations (Pixels)	Mean Value of Colored Pixels in Double Nest Occupations (Pixels)	Values of Significance
1	11,272	21,439	*p* < 0.01
3	2373	5172	*p* < 0.01
5	603	1425	*p* < 0.01

**Table 2:** Detection performances, reached during the setup procedure (step A), reported for each mean temperature shift investigated (i.e., 1, 3, and 5 °C). Performances are shown in the table in terms of sensitivity and specificity. Furthermore, the corresponding cutoff level, in terms of number of colored pixels, that allows to classified a multiple occupation of the nest is also reported.

Shifts of the Mean Floor Temperature (∆*t*-°C)	Sensitivity (%)	Specificity (%)	Cut-off Level (Pixels)
1	80.1	87.3	13,509
3	80.0	90.9	2877
5	80.1	91.5	796

**Table 3:** Shapes and geometric features of the templates used to perform the image pattern recognition procedures in the following field tests. For each geometric feature, the corresponding mean floor temperature shift investigated is reported (i.e., 1, 3, and 5 °C). Furthermore, the results obtained performing a Student’s *t*-Test procedure on data collected in terms of means, standard error (S.E.), confidence intervals and significance of the means, are also shown in the table.

Shifts of the Mean Floor Temperature (∆*t*-°C)	Template Shapes	Geometric Futures	Values of the Mean and S.E. (Pixels)	Confidence Intervals at 95% (Pixels)	Values of Significance
1	ellipse	major axis	158 ± 4	151–166	*p* < 0.01
1	ellipse	minor axis	83 ± 3	76–89	*p* < 0.01
3	triangle	height	68 ± 1	65–71	*p* < 0.01
3	triangle	base	47 ± 1	46–49	*p* < 0.01
5	triangle	height	43 ± 1	42–44	*p* < 0.01
5	triangle	base	33 ± 1	32–34	*p* < 0.01

**Table 4:** Detection performances, reached during the setup procedure (step B), reported for each mean temperature shift investigated (i.e., 1, 3, and 5 °C). In this elaborations, a defined cutoff level was used in accordance with the results reported in Table 2 and image pattern recognitions were performed using a specific template, according to results reported in Table 3. Performances are shown in the table in terms of sensitivity and specificity.

Shifts of the Mean Floor Temperature (∆*t*-°C)	Sensitivity (%)	Specificity (%)	Image Pattern Recognition Templates (Shape and Geometric Features in Pixels)
1	57.4	89.0	ellipse (158 × 83)
3	70.3	81.8	triangle (68 × 47)
5	72.3	90.7	triangle (43 × 33)

**Table 5:** Performances of the sensor in the detection of a multiple occupation of the nest during an oviposition.

Results Classification	Positive	Negative	Total
True	45	125	170
False	6	2	8
Total	51	127	178

**Table 6:** Performances of the sensor in the detection of a double or triple occupation of the nest during an oviposition.

Type of Occupation of the Nest	Results Classification	Positive	Negative	Total
Double	True	31	129	160
	False	7	11	8
	Total	38	140	178
Triple	True	4	164	168
	False	9	1	10
	Total	13	165	178
